# App-Based Habit Building Reduces Motivational Impairments During Studying – An Event Sampling Study

**DOI:** 10.3389/fpsyg.2020.00167

**Published:** 2020-02-07

**Authors:** Marco Stojanovic, Axel Grund, Stefan Fries

**Affiliations:** Department of Psychology, Bielefeld University, Bielefeld, Germany

**Keywords:** habit formation, motivational interference, self-control, learning, app intervention, event sampling

## Abstract

In this app-based event sampling study, we observed the intentional formation of new study habits. A sample of 91 university students defined individual study habits and logged data over 6 weeks on motivational conflict, motivational interference (MI) and automaticity of behavior after each habit repetition using an app on their phone. The app was specifically created for this study and gave feedback on habit automaticity. A total of *N* = 2,574 habit repetitions have been generated and were analyzed using multilevel modeling. The results suggest that (1) app-based intentional habit building works, as automaticity of behavior could be predicted by habit repetition, (2) motivational impairments during studying can be reduced by building habits, as want conflicts and MI decreased with automaticity, and (3) trait self-control supports studying indirectly by fostering habit building rather than directly by suppressing impulses during the activity, as self-control predicted automaticity, but not motivational impairments during the habit execution. The effect of self-control on automaticity of the new study habit was fully mediated by the general automaticity of the students’ other study habits (general study habit strength). This study showcases an app-guided genesis of new study habits and its beneficial motivational effects for learning behavior.

## Introduction

When we navigate through our everyday life, we act habitually in about 50% of the time (Wood et al., 2002). That means we make unconscious, quick choices about what we do and act it out automatically without much effort (Gardner et al., 2016). When a habit is instigated, one is guided by automized behavior and less by deliberate intention (Ouellette and Wood, 1998). Habits are a strong force that can harm (e.g., a TV habit fostering procrastination) or help us (e.g., a writing habit for term papers). In this study, we intended to apply the force of habit to learning behavior in order to tame well-known motivational problems through habitual automaticity.

It is a common situation: A student sits at his/her desk to study for an upcoming exam. At the same time however, thoughts about his/her friends watching a movie in the cinema right now intrude his/her thinking and destabilize the learning process. He/she is experiencing a motivational conflict (Fries and Dietz, 2007) that can come up whenever there are at least two conflicting motivational tendencies active in a person’s mind. Grund et al. (2015a) distinguish motivational conflicts as want conflicts (WC) (i.e., wanting to do something else than the focal activity) or should conflicts (i.e., feeling that one should be doing something else than the focal activity). The aforementioned student is experiencing a WC as he/she is sitting at his/her desk wishing to be with his/her friends and trying to study. If he/she went to the cinema with his/her friends, however, and experienced a feeling that he/she should be studying, he/she would have experienced a should conflict.

Studying can indeed be an arduous endeavor. While engaged in study related activities, students in an experience sampling study reported wanting to do something else (i.e., WC) in 63.1% of the time^1^ (Grund et al., 2015b). This high prevalence of motivational conflicts while studying should be of concern for everyone who is interested in good education – on an individual and societal level – because motivational conflicts cause motivational interference (MI) (e.g., Fries and Dietz, 2007). MI is defined as “the process by which incentives of conflicting options destabilize the current activity” (Hofer and Fries, 2016, p. 445) and manifests itself in bad mood, distractibility, thoughts about the alternative, task switching and low persistence (Hofer and Fries, 2016). It leads to an array of undesired study-related outcomes. MI is associated with impairments in self-regulation (Fries et al., 2008) and learning (Brassler et al., 2015), lower well-being (Riediger and Freund, 2008; Grund et al., 2015a) and lower academic and social adaption (Grund et al., 2014). Galla and Duckworth (2015, study 2) found that students with stronger study habits reported having less MI in an imagined study-leisure conflict.

In the present paper, we want to investigate whether we can help students to build new study habits and, if so, whether such study habits are helpful for experiencing less MI in the real world. In addition to that, we explore the role of self-control in relation to habit building and MI. As self-control is associated with stronger desirable habits (e.g., Galla and Duckworth, 2015) and less MI while studying (e.g., Grund and Fries, 2014), it can be considered a crucial personality variable influencing both habit forming and the occurrence of MI.

### Habits

A habit is a behavioral pattern (e.g., preparing for an exam using a fixed routine) learned through context-dependent repetition (e.g., studying at one’s desk before dinner over and over again in order to prepare for exams; Ouellette and Wood, 1998; Lally et al., 2010; Gardner et al., 2011). This general habit definition can be adapted to behavior in different domains like exercise, eating or, as in this case, studying. Habits in general are strongly connected to actual behavior. The correlations between habit strength and respective behaviors related to nutrition, healthy/unhealthy diet and physical activity/inactivity are in the range of *r* = 0.41 – 0.44 (Gardner et al., 2011). Study habits in particular are associated with less MI while studying, a greater ability to study under difficult circumstances, higher classroom engagement, higher homework completion (Galla and Duckworth, 2015) and a higher GPA (Credé and Kuncel, 2008; Galla and Duckworth, 2015).

Study habits can be seen as a subclass of ordinary habits according to the above-mentioned definition, but they differ significantly from very simple and static habits like a running habit with a fixed set of consecutive steps. Study habits are more like flexible frameworks that are applied to different learning contents. A student might run the same route every time within his/her running habit, but he/she will not learn the same lecture over and over again within his/her study habit. This point will be explained in more detail in the following section.

When we see a student sitting at his/her desk studying, how can we determine whether his/her behavior is habitual or not? Traditionally, one would measure the habit strength simply by the frequency of the behavior in the past (Triandis, 1977). However, even when frequency of behavior is controlled, it can be experienced more or less habitual (Verplanken, 2006). For that matter, in modern habit research, automaticity became the central distinguishing criterion for determining whether or not a behavior can be seen as habitual (Gardner, 2012). More importantly, in contrast to frequency, automaticity can be used to track the development of new habits (Lally et al., 2010).

The study of Lally et al. (2010) is a key study for habit research, because it’s the only one to have tracked the development of entirely new habits over a significantly long timespan (12 weeks) in the noisy real world environment and shows that habits can be built intentionally. The development of new habits can be described by a quadratic function. New habits tend to have steep increases in automaticity in the beginning and increase less and less in automaticity with each repetition until reaching an asymptote after a strongly varying range of time (18–254 days). This asymptote can be imagined as a habit’s glass ceiling. The high variance in individual automation can partly be explained by the fact that each participant chose her/his own habit to develop in the field of healthy eating, drinking, or exercise. Thus, the pool of the observed habits ranges from rather easy and simple (e.g., “eating a piece of fruit with lunch”) to relatively difficult and more complex (e.g., “running for 15 min before dinner”), which is resembled by the present study data.

### The Flexibility of Study Habits

**FIGURE 1:**
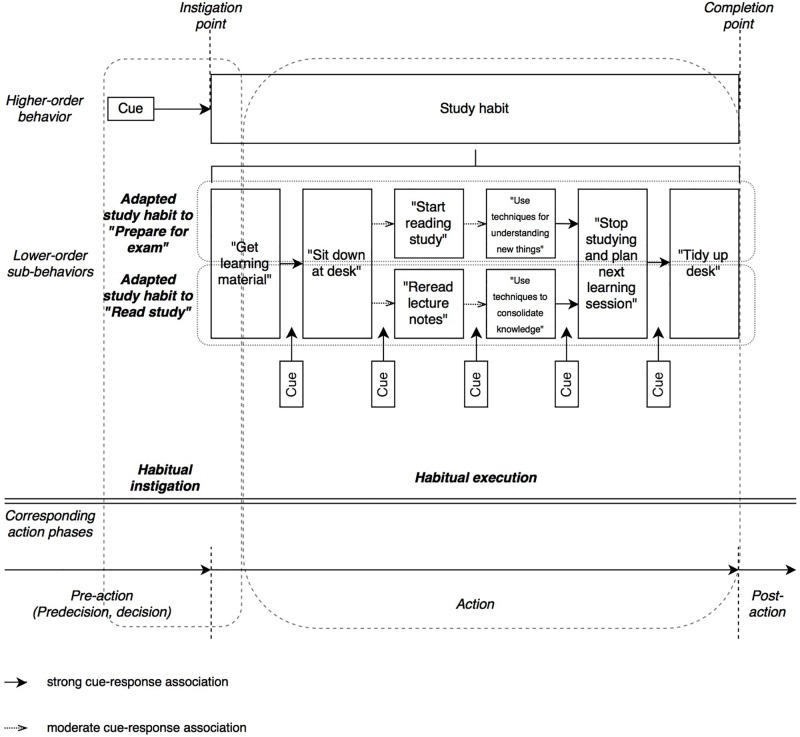
A complex and flexible study habit that is adapted to different learning contents (adapted from Gardner et al., 2016).

The fact that study habits are behavioral patterns that are adapted to different contents make them flexible und useful on the one hand, but more difficult to build and fragile on the other hand. Gardner et al. (2016) describe a running habit by decomposing the higher-order act of “going for a run”, that is started by a certain cue (*habit instigation*), into lower-order sub-behaviors that are cued sequentially (*habit execution*; e.g., “put on sneakers”, “leave the house”, “walk to park” etc.), leading the person through the habit from beginning to end. Such a habit can reach a high absolute asymptote in automaticity because the cue-response chaining of the sub-behaviors stays the same. See Figure 1 to compare this simple running habit to a flexible study habit that is applied to two different learning contents: Preparing for an exam and reading a study.

As can be seen in Figure 1, the study habit is comprised of a mix of strong and moderate cue-response connections, while the sub-behaviors of the running habit would only have strong ones. Study habits are like flexible frameworks that can be adapted to different contents (e.g., preparing for an exam or read a study), but that lowers the absolute maximum of the possible automaticity asymptote, because not every cue-response is activated with every habit repetition when performing it. However, when a simple, static habit like running is performed, every cue-response pair of the sub-behaviors is activated resulting in a faster automation and a more robust habitual execution. Furthermore, due to their flexible nature, study habits are less sequential to the extent the content demands it. While studying, a student might realize that he/she needs another book in order to continue or he/she might stop reading to google unknown words from time to time.

Nevertheless, even a flexible and complex study habit can become more automatic with each repetition because many cue-response sub-behaviors stay the same independently of the learning content. Especially when starting one’s study habit, often content invariant behavior such as preparing the workplace, is needed, which makes the connection between the habit-instigation-cue and the first sub-behavior more automizable. So it is likely that the instigation of study habits will become easier even if they are generally more difficult to automize due to high flexibility in habit execution. In our example (see Figure 1), the habit starts and ends in the same way, providing a stable study habit framework for different learning tasks. Furthermore, even less stable cue-response sub-behaviors (e.g., “summarize a text passage” within “using techniques for understanding new things” cued by having finished a passage), will get automized, and thus adding to the overall automaticity of the study habit, even though it might not be applied in every habit repetition.

In summary, study habits differ in one crucial point from other habits: They are constantly adapted to new learning contents, making them more useful, complex and flexible, but also more fragile as not every cue-response pair is activated in every habit repetition. Nevertheless, performing a similar learning pattern in the same context over and over again (i.e., building a study habit) should strengthen the habit instigation as well as parts of habit execution, resulting in a stable, yet malleable behavioral framework for learning. Hypotheses H1a-b aim at replicating the asymptotic automaticity growth curve reported by Lally et al. (2010) for the formation of new study habits.

**H1a**: Automaticity will increase with habit repetitions.

**H1b**: The automaticity growth will decrease asymptotically over habit repetitions.

### Habits Reduce Motivational Interference

The Rubicon model (Heckhausen and Gollwitzer, 1987; Boekaerts and Corno, 2005) is widely used in the field of psychology to describe the overall process of self-regulated learning with four sequential phases: Pre-decisional (choosing an activity), pre-actional (planning the activity), actional (performing the activity), and post-actional (evaluate the result). Having chosen an activity and entering the pre-actional planning phase is considered “crossing the Rubicon” in this model, meaning that one is now committed to the chosen activity and entirely focused on it. The concept of MI (Fries and Dietz, 2007) waters down the models’ strict separation of contemplating about other activities of the pre-decisional phase and the pursuit of a focal activity in the pre-actional and actional phase by showing that thoughts about unchosen activities can impact focal activities. The stronger the motivation is for the unchosen activity, the stronger the MI (Fries et al., 2008; Grund, 2013). MI happens post-decisional. There *is* a bridge leading back to all the possibilities and thinking about it can destabilize the activity at hand.

A central finding in habit research is that performing habitual behavior requires much less intention than non-habitual behavior (Ouellette and Wood, 1998; Webb and Sheeran, 2006; Limayem et al., 2007). With this waning connection of intention and actual behavior, the transitions between the sequential steps of choosing, planning and performing become more smooth and automatic as soon as the instigation cue is present that triggers a habit. A lack of awareness, that, among others, refers to starting a habit (*habit instigation*) without realizing it immediately, is a facet of habit automaticity (Verplanken and Orbell, 2003). One slips into habitual behavior rather than having to intentionally decide for or against it every time. Habits make passing the Rubicon easier. In the Rubicon model, the performance of the sub-behaviors of a habit (*habit execution*) refers to the actional phase.

Performing a habit means performing an automized task. There is evidence that this should “free the memory” for other thoughts that are unrelated to the focal activity (Wood et al., 2002). One could think this would actually increase the likelihood of motivational conflicts as there is now space for new, unrelated thoughts. However, study habits are, as discussed above, rather flexible and complex. They give a frame for the activity, but have constantly changing contents (i.e., a student learns new things every time, but in the same manner). Furthermore, empirical evidence addressing this question finds the opposite: Study habits correlate negatively with MI (Galla and Duckworth, 2015, study 2). However, the data is merely correlational, obtained from a cross-sectional online study with an imagined motivational conflict. The longitudinal event sampling design with entirely new study habits allows causal inferences about habit strength and MI in a noisy real life setting.

A habit is easier to start when one is in the right context (habit instigation in the pre-decisional phase) and easier to perform (habit execution in the actional phase) than non-habitual behavior as less intention is needed for automized habitual behavior. We hypothesize that automaticity helps in shielding the focal activity from alternative options, which should result in less motivational WCs and less MI. The hypothesis only includes WCs as this type of motivational conflict is far more prevalent while studying (63.1% of the time) than should conflicts (19.0% should and WCs mixed, 3.1% pure should conflicts; Grund et al., 2015b).

**H2a**: The more automized a study habit is, the less WCs are experienced during its performance.

**H2b**: The more automized a study habit is, the less intense are indicators of MI during its performance.

### The Role of Self-Control in Habit Building

Self-control is the ability to resist or modify behavioral tendencies (e.g., impulses; Tangney et al., 2004). There are two paths, direct and indirect, in which self-control can have an effect on the process of forming a new study habit. The direct path is the main effect of the exertion of self-control during the habit repetition. The indirect path is a mediation via previously built study habits.

#### The Direct Path

The more automatic a contextually triggered behavioral pattern is (i.e., the stronger a habit is), the less self-control resources are needed to regulate that behavior (Neal et al., 2013). However, meta-analytical results show that self-control is strongly connected to the automatization of desirable behaviors (de Ridder et al., 2012). In the beginning of the habit building process, the new behavior is not yet automized and performing this new pattern of behavior might feel a little odd. At this point, it is very valuable to be able to exert self-control in order to resist impulses to stop the new behavior before having finished. The next habit repetition is going to be a little easier as it will be more automized and less self-control should be needed to continue building the new habit. A positive correlation between self-control and having stronger study habits has already been shown (Galla and Duckworth, 2015, study 2). For the process of building new study habits, we expect to find this positive association between self-control and habit strength as well. We hypothesize the following.

**H3a**: The higher self-control, the more automaticity is experienced during the habit building process.

#### The Indirect Path

de Ridder et al. (2012, p. 90) point out, that surprisingly “the effects of self-control on automatic behaviors were consistent across both desired [*r* = 0.36] and undesired behaviors [*r* = −0.40] and were overall the largest effect-sizes in our entire meta-analysis.”. It seems to be the case that highly self-controlled people are very good at building good habits and bypassing bad ones, allowing them to perform adaptive behavior or avoid maladaptive behavior with less effort, which would explain the repeatedly found negative correlation between self-control and its actual usage (Hofmann et al., 2012; Galla and Duckworth, 2015; Grund and Carstens, 2019). Furthermore, Galla and Duckworth (2015) found that habits mediated the effect of self-control on desirable outcomes like GPA and college persistence. As pointed out above, study habits are likely to be complex patterns that contain different parts that might be rather fixed and content-invariant or flexibly adapted to new contents. When building a new study habit, the learner does not start with a blank slate. There is a whole arsenal of useful learning practices and previously used patterns that can be understood as building blocks for the habit to be formed. When a learner has generally strong study habits, he/she has a very broad and highly automized range of these “pre-automized” building blocks that can be used to put together to a new study habit. Naturally, this should increase automaticity of the new habit. Someone who has strong habits for reading literature, summarizing lectures, learning vocabulary, and preparing for exams should be more likely to successfully build a writing habit for example than someone who has overall weak study habits. Taking into account the strong tendency of highly self-controlled people to automate desirable behavior (i.e., form good habits), strong general study habits can be understood as a domain specific form of manifested self-control. Thus, next to the direct effect of self-control on habit building, we expect an indirect effect of self-control on automaticity via general study habit strength, which should result in a partial mediation (H3c). For that, significant main effects of self-control (H3a) and general study habit strength (H3b) should emerge. We hypothesize the following.

**H3b**: The higher general study habit strength, the more automatic the habit performance will be.

**H3c**: General study habit strength partially mediates the effect of self-control on automaticity.

Concerning MI, students with higher self-control report having less MI (Galla and Duckworth, 2015, study 2) and less WCs (Hofer et al., 2012; Grund and Fries, 2014; Grund et al., 2015a). The exertion of self-control in order to shield the focal activity from destabilizations like thoughts about alternative activities or impulses to stop the learning process should reduce MI during studying. We hypothesize the following.

**H3d**: The higher self-control, the less WCs are experienced during habit performance.

**H3e**: The higher self-control, the less MI is experienced during habit performance.

## Materials and Methods

### Participants

A total of *N* = 91 university students (*M_*age*_* = 22.3, *SD_*Age*_* = 4.9, range from 18 to 50 years; 79.1% female) participated in return for course credit. Participants that showed high compliance (at least 20 habit repetitions) took part in an additional lottery for a new iPad worth 500 Euros (∼ 600 US dollars). Participants who did not finish the pretest or who had not at least one habit repetition were excluded from the data analysis. The participants were recruited via announcements in undergraduate psychology lectures of two German universities. During these announcements, a short presentation on how habits can be useful for studying was given by the first author.

The sample provides sufficient statistical power for our analyses. According to Maas and Hox (2005) multilevel models with medium effect sizes (0.3; Cohen, 1988) and Level 2 sample sizes of over 50 (i.e., participants in the present case) with at least 5 Level 1 observations (i.e., habit repetitions per participant) can be estimated accurately. For our non-hierarchical mediation analysis, assuming a large effect of the predictor on the mediator and a medium effect of the mediator on the dependent variable, satisfactory power is reached with a Level 2 sample size of 67 (Fritz and MacKinnon, 2007).

### Procedure and Measures

The participants tracked their individual habit building process over 6 weeks with an iPhone app that was specifically created for this study.

#### Onset

##### App installation

The app was either installed via an email link or directly from the first author’s computer to the iPhones of the participants. The app was not publicly available. After installation, the participants were prompted to create a user account with an individual, anonymous participant ID. This ID was used to store the data for each participant.

##### Mini-tutorial

After logging in for the first time, a short tutorial was shown that explained the value of study habits and prepared the participants for the next steps.

#### Pretest

Having completed the tutorial, the participants were presented the home screen that prompted them to answer a personality questionnaire (i.e., the pretest) which took about 20 min to complete, as there were other constructs assessed that are not relevant for this paper. All items were answered with a slider on a 11-point scale (from *0* = *doesn’t apply at all* to *10* = *applies perfectly*) and were shown one at a time. The participants could start the pretest anytime they wanted.

##### Self-control

Trait self-control was measured with the German version (Bertrams and Dickhäuser, 2009) of the 13-item Brief Self-Control Scale (Tangney et al., 2004). The scale asks for general indicators of the ability to exert self-control (e.g., “I am good at resisting temptation.”). Higher scores mean higher self-control. Cronbach’s α for this sample was good with 0.81.

##### General study habit strength

To measure the general study habit strength, the Self-Report Habit Index (SRHI; 12 items; Verplanken and Orbell, 2003) was translated into German and adapted by the first author (e.g., “Studying for university/school is something I do frequently.”). Cronbach’s α for this sample was high with 0.94.

#### Habit Definition

After the pretest was completed, the participants got back to the home screen and were prompted to define their new study habit. The app guided the participants through a habit definition process that consisted of four steps: (1) What, (2) When, (3) How long, (4) Goal. Each step had a short instruction, two constraints, examples, and a text field in which the participants entered their respective answers for each step. This habit definition process was adapted from Lally et al. (2010). At the end, participants were asked to set a reminder for their newly defined habit.

##### What

The participants were asked to describe their new habit here: *What* shall be the new habit? The two constraints were: *It must be for university* and *It must be a new habit.* To clarify this step, the participants were given examples like *reading relevant literature* and *summarizing a lecture*.

##### When

The participants were asked to describe *when* they wanted to perform their new habit. The two constraints were: *It must be performed daily* and *It must be pegged to another daily activity.* The latter constraint was supposed to connect the new habit to a daily recurring environmental cue that could then, after a some repetitions, trigger the habit effectively (Ouellette and Wood, 1998). To clarify this step, the participants were given examples like *after breakfast, after coming home from university*, and *before brushing teeth*.

##### How long

The participants were asked to describe *how long* it would approximately take to perform their new daily habit. The two constraints were: *It must be at least 10 min* and It must *not be more than 30 min.* To clarify this step, the participants were given examples like *10–15 min* and *20 min*.

##### Goal

The participants were asked to set a *goal* for the daily habit repetition. The two constraints were: *It must be attainable within the previously defined time frame* and *It must be measurable.* To clarify this step, the participants were given examples like *read 5 pages* and *summarize ONE whole lecture*.

##### Set daily habit reminder

The participants were asked to set a time at which they wanted to be reminded of their habit daily. However, setting a reminder was not mandatory and the participant could end the habit definition process without setting a reminder. It was tracked whether or not a participant chose to set a reminder. Eighty six (94.5%) of the 91 participants set a reminder as recommended.

After the last step of this section, the app saved the current date as the start date of the study for the respective participant and the event sampling period began. Each participant could from now on see her/his defined habit in the settings.

#### Event Sampling

While the first three steps (onset, pretest, and habit definition) were only performed once, participants were instructed to perform this fourth step daily for 6 weeks. All items, apart from the goal attainment item, were answered with a slider on a 11-point scale (from *0* = *doesn’t apply at all* to *10* = *applies perfectly*) and were shown one at a time. Whenever no habit data had been entered at a given day, the home screen prompted the participants to enter data after having finished their individual study habit.

##### Check if habit is completed for the day

The participants were asked to confirm that they had finished their study habit for the current day. It was not possible to enter data without confirming.

##### Motivational interference

MI was measured with five items that cover the five facets *mood* (“I was annoyed by my habit.”), *distractibility* (“My thoughts constantly digressed.”), *thoughts about alternatives* (“From time to time I thought about other things I let slide.”), *task switching* (“I switched between different activities.”), and *persistence* (“It was difficult to finish my habit.”). The items for *mood*, *distractibility*, *thoughts about alternatives*, and *task switching* were adapted from Grund et al. (2014) and the item for *persistence* was adapted from Brassler et al. (2015).

##### Habit automaticity

Habit strength was measured via 6 adapted automaticity items from the SRHI (e.g., “My habit is something I do automatically.”; Verplanken and Orbell, 2003; Lally et al., 2010). For each data entry, the app presented an automaticity index between 0 and 100 (the average of the habit repetition’s automaticity score multiplied by 100) that could be shown anytime by tapping on a chart icon and thus depicted the progress in habit strength.

##### Motivational want conflict

To measure if participants experienced a WC during the study habit, they were asked if they *wanted* to do something else while performing their habit (“During my habit I WANTED to do something else.”; Grund et al., 2015a).

##### Degree of goal attainment

The participants were asked to rate the degree to which they reached their previously defined daily habit goal for the current habit repetition on a scale from *0–100%* (“How much percent of your set habit goal did you attain today?”).

### Data Analysis

Through the event sampling process, hierarchical data were obtained where habit repetitions (Level 1) are nested within persons (Level 2). To test if there is substantial Level 2 variance so multilevel modeling would make sense, intraclass correlation (ICC) analyses were conducted for each dependent variable. All ICC analyses were conducted with the 57 participants who finished at least 21 habit repetitions. Then, using IBM SPSS 20, growth curves were modeled with multilevel regressions (Field, 2013) to test H1a-b, H2a-b and H3d-e. Please note that Models 3 and 4 were created to replicate the pattern of the mediation assumed in hypotheses H3a-c (self-control predicts automaticity, but loses predictive power when general study habit strength is added as a predictor) while controlling for habit repetitions. The mediation itself is tested on person level using the SPSS marco PROCESS v3 (Hayes, 2017) as described in the last paragraph of this section. The growth curves describe the development of the dependent variables automaticity, WCs and MI over time (i.e., habit repetitions). Maximum likelihood parameter estimation and an unstructured covariance matrix for random effects were used in all growth models. All models were specified with three random parameters: Random intercepts (*u*_0_), random slopes (*u*_1_) and the covariance of the random intercepts and the random slopes [COV(*u*_0_,*u*_1_)]. The parameters *u*_0_ and *u*_1_ are Level 2 (person) residual terms and represent individual deviations from the average intercept respectively from the average slope of the whole sample. As *u*_0_ and *u*_1_ model Level 2 variability, they are not estimated as coefficients but tested for significant variance. In all growth curve models, time was modeled with the habit repetition variable, meaning that for example timepoint t = 5 corresponds to the point in time during the fifth habit repetition. All coefficients in our analyses, in the growth curves as well as in the mediation analysis, are unstandardized.

Model 1 (H1a; Equation 1) predicts automaticity at time t for person p with a random intercept *b*_0__,p_, which is the average intercept of the sample *b*_00_ plus the individual deviation from that intercept *u*_0__,p_ (Equation 2), plus the individual slope *b*_1__,p_, which is the average slope for the effect of habit repetition (i.e., time) of the whole sample *b*_10_ plus the individual deviation from that slope *u*_1__,p_ (Equation 3), times habit repetition plus error ε_*t,p*_.

(1) $${\text{Automaticity}}_{\text{t},p}=b_{0,\text{p}}+b_{1,\text{p}}\,\mathrm{Habit}\,{\mathrm{repetition}}_{\text{t},p}+\varepsilon_{\text{t},\text{p},\cdot }$$

(2) $$b_{0,\text{p}}=b_{00}+u_{0,\text{p},\cdot }$$

(3) $$b_{1,\text{p}}=b_{10}+u_{1,\text{p},\cdot }$$

In Model 2 (H1b), a quadratic trend was added to the automaticity growth curve by entering habit repetition squared (habit repetition sq) as an additional Level 1 predictor to Equation 1 resulting in Equation 4. *b*_0__,p_ and *b*_1__,p_ are the same as in Model 1.

$${\text{Automaticity}}_{\text{t},p}=b_{0,\text{p}}+b_{1,\text{p}}\,\mathrm{Habit}\,{\mathrm{repetition}}_{\text{t},p}$$

(4) $$+b_{2}\mathrm{Habit}\mathrm{repetition}{\mathrm{sq}}_{\text{t},\text{p}}+\varepsilon_{\text{t},\text{p}}\cdot$$

In Model 3 (H3a), self-control was added as a Level 2 fixed effect to Model 2, which resulted in a modification of the random intercept *b*_0__,p_. The intercept in this model is estimated as in Equation 2 plus the effect of self-control: *b*_01_ times self-control. Model 3 can be represented by Equation 4 with the modified intercept as specified in Equation 5.

(5) $$b_{0,\text{p}}=b_{00}+b_{01}\,\text{Self}\,{\text{control}}_{\text{p}}+u_{0,\text{p},\cdot }$$

In Model 4 (H3b-c), general study habit strength was added as a Level 2 fixed effect to Model 3, which resulted in a modification of the random intercept *b*_0__,p_. The intercept in this model is estimated as in Equation 5 plus the effect of general study habit strength: *b*_02_ times general study habit strength. Model 4 can be represented by Equation 4 with the modified intercept as specified in Equation 6.

$$b_{0,\text{p}}=b_{00}+b_{01}\,\mathrm{Self}\,{\mathrm{control}}_{\text{p}}$$

(6) $$+b_{02}\,\mathrm{General}\,\mathrm{study}\,\mathrm{habit}\,{\mathrm{strength}}_{\text{p}}+u_{0,\text{p},\cdot }$$

Model 5 and Model 7 have the same equations as Model 1 but with WC (Model 5) and MI (Model 7) as dependent variables. In Model 6 (H2a) and Model 8 (H2b), the individual automaticity scores from the last habit repetition (automaticity_*t–*__1_) were added as a Level 1 predictor, resulting in Equation 7 and Equation 8. *b*_0__,p_ and *b*_1__,p_ are the same as in Model 1.

$$\mathrm{Want}\,{\mathrm{conflict}}_{\text{t},\text{p}}=b_{0,\text{p}}+b_{1,\text{p}}\,\mathrm{Habit}\,{\mathrm{repetition}}_{\text{t},p}$$

(7) $$+b_{2}\,{\text{Automaticity}}_{\text{t}-1,\text{p}}+\varepsilon_{\text{t},\text{p},\cdot }$$

$$\mathrm{Motivational}\,{\mathrm{interference}}_{\text{t},\text{p}}=b_{0,\text{p}}+b_{1,\text{p}}\,\mathrm{Habit}\,{\mathrm{repetition}}_{\text{t},p}$$

(8) $$+b_{2}\,{\text{Automaticity}}_{\text{t}-1,\text{p}}+\varepsilon_{\text{t},\text{p},\cdot }$$

See Table 1 for the models predicting automaticity (Model 1 – 4) and Table 2 for the models predicting WCs and MI (Model 5 – 8). All other models we tested are based on the models described above and can be derived by adding the tested predictor to the respective model.

**TABLE 1:** Multilevel regressions of automaticity on habit repetition, habit repetition squared, self-control and general study habit strength.

	**Automaticity**											
**Parameter**	**Model 1 (H1a)**			**Model 2 (H1b)**			**Model 3 (H3a)**			**Model 4 (H3b-c)**		
				
	**Estimate**	***SE***	**95% CI**	**Estimate**	***SE***	**95% CI**	**Estimate**	***SE***	**95% CI**	**Estimate**	***SE***	**95% CI**
**Fixed effects**												
Intercept (*b*_00_)	2.241***	0.192	1.860, 2.622	2.000***	0.194	1.615, 2.385	0.033	0.615	−1.189, 1.254	0.103	0.586	−1.060, 1.266
Level 1												
Habit repetition (*b*_10_)	0.074***	0.008	0.058, 0.091	0.113***	0.009	0.095, 0.131	0.113***	0.009	0.095, 0.131	0.113***	0.009	0.095, 0.132
Habit repetition sq (*b*_2_)				−0.001***	<0.001	−0.0013, −0.0008	−0.001***	<0.001	−0.0013, −0.0008	−0.001***	<0.001	−0.0013, −0.0008
Level 2												
Self-control (*b*_01_)							0.428***	0.128	0.174, 0.682	0.108	0.158	−0.204, 0.421
General study habit strength (*b*_02_)										0.345***	0.108	0.131, 0.559
**Random effects**												
Random intercept (VAR *u*_0_)	3.192***	0.495	2.355, 4.326	3.216***	0.498	2.374, 4.358	2.843***	0.445	2.092, 3.682	2.591***	0.405	1.908, 3.521
Cov. rand. intercept, rand. slope (COV *u*_0_, *u*_1_)	−0.030*	0.015	−0.0593, −0.0004	−0.031*	0.015	−0.0612, −0.0013	−0.029*	0.015	−0.0583, −0.0005	−0.030*	0.014	−0.0575, −0.0030
Random slope (VAR *u*_1_)	0.004***	<0.001	0.003, 0.006	0.004***	<0.001	0.003, 0.007	0.004***	<0.001	0.003, 0.007	0.004***	<0.001	0.003, 0.007

All estimated coefficients are unstandardized. Variables as specified in the model equations are in parentheses. CI, confidence interval; Habit repetition sq, habit repetition squared; VAR, variance; COV, covariance; Cov. rand. intercept, rand slope, covariation of the random intercept and the random slope. *p < 0.05, ***p <0.001.

**TABLE 2:** Multilevel regressions of want conflict and motivational interference on habit repetition and automaticity_*t–*__1_.

**Parameter**	**Model 5**			**Model 6 (H2a)**			**Model 7**			**Model 8 (H2b)**		
				
	**Want conflict**						**Motivational interference**					
		
	**Estimate**	***SE***	**95% CI**	**Estimate**	***SE***	**95% CI**	**Estimate**	***SE***	**95% CI**	**Estimate**	***SE***	**95% CI**
**Fixed effects**												
Intercept (*b*_00_)	5.543***	0.248	5.050, 6.035	5.790***	0.269	5.257, 6.324	3.771***	0.179	3.416, 4.126	4.067***	0.196	3.678, 4.456
Level 1												
Habit repetition (*b*_10_)	−0.049***	0.009	−0.066, −0.032	−0.029***	0.008	−0.046, −0.012	−0.049***	0.007	−0.063, −0.036	−0.046***	0.007	−0.060, −0.033
Automaticity_*t–*__1_ (*b*_2_)				−0.200***	0.041	−0.279, −0.119				−0.100***	0.030	−0.157, −0.042
**Random effects**												
Random intercept (VAR *u*_0_)	4.961***	0.810	3.603, 6.831	5.027***	0.844	3.617, 6.987	2.559***	0.428	1.843, 3.553	2.656***	0.455	1.899, 3.716
Cov. rand. intercept, rand. slope (COV *u*_0_, *u*_1_)	−0.048*	0.020	−0.088, −0.009	−0.048*	0.019	−0.085, −0.010	−0.051***	0.013	−0.077, −0.026	−0.054***	0.014	−0.080, −0.027
Random slope (VAR *u*_1_)	0.004***	<0.001	0.002, 0.006	0.003***	<0.001	0.002, 0.005	0.002***	<0.001	0.002, 0.004	0.002***	<0.001	0.001, 0.004

All estimated coefficients are unstandardized. Variables as specified in the model equations are in parentheses. CI, confidence interval; Automaticity_*t*–__1_, automaticity value of the previous habit repetition; VAR, variance; COV, covariance; Cov. rand. intercept, rand slope, covariation of the random intercept and the random slope. *p < 0.05, ***p < 0.001.

To test if general study habit strength mediates the effect of self-control on automaticity (H3a-c), a Level 2 mediation analysis was conducted using the SPSS macro PROCESS v3 (see Hayes, 2017). As general study habit strength and self-control are person level variables, the participants’ mean automaticity score was used as the dependent variable in order to be able to conduct a mediation analysis on person level. The mediation analysis contains four steps. In the first step, a significant relationship between the predictor (self-control) and the dependent variable (automaticity) needs to be shown. In the second step, a significant relationship between the predictor (self-control) and the mediator (general study habit strength) needs to be shown. In the third step, a significant relationship between the mediator (general study habit strength) and the dependent variable (automaticity) needs to be shown in the presence of the predictor (self-control). In the fourth step, a meaningful reduction of the effect of the predictor (self-control) on the dependent variable (automaticity) needs to be shown in the presence of the mediator (general study habit strength). The direct and indirect effects of the mediation analysis were then estimated via bootstrapping with 5000 samples.

## Results

### Preliminary Findings

The 91 participants generated a total of *N* = 2,574 habit repetitions with an average of *M* = 28.29 (*SD* = 17.88) habit repetitions. The dataset did not contain any missing values. *n* = 57 participants (62.6%) completed at least 21 habit repetitions and *n* = 27 (29.7%) completed at least 42 habit repetitions. Twenty one participants continued to enter data above the timeframe of 6 weeks they were asked to log data. The participant with the most habit repetitions logged 69 measurement points. These habit repetitions are included into the data analysis. A total of *n* = 1,953 (78.7%) of the habit repetitions have been done without pause, i.e., the day after the last repetition, *n* = 336 (13.5%) were done with a pause of 1 day, *n* = 97 (3.9%) were done with 2 days pause and the remaining *n* = 97 (3.9%) repetitions were done with three or more days pause. The average degree of goal attainment, which was measured for each repetition, was 83.4% (*SD* = 21.46) with a median of 91%. Participants reached 100% (vs. 0%) of their set habit goal in 39.9% (vs. 0.3%) of the cases. This indicates that the study related activities during the habit repetitions yielded highly productive learning behavior.

The individually defined study habits were categorized according to the main learning activity (frequencies and examples in parentheses): Revision of lectures/seminars (35.2%, e.g., “Revise Statistics lecture”), reading (18.7%, e.g., “Read literature about biopsychology”), writing (13.2%, e.g., “Write exam paper”), rehearsal (9%, e.g., “Repeat flashcards about brain anatomy for 10 min”), a mix of the categories (6.6%, e.g., “Read/write/research regularly for my bachelor thesis”), other (17.6%, e.g., “Practice strength and stabilization exercises for the gymnastics course,” “Watch English YouTube videos to improve my English and understand texts better.”).

### Habit Formation

The first two hypotheses (H1a-b) aimed at replicating the automaticity growth curve of newly developing habits as found in the study by Lally et al. (2010), which is characterized by a steep automaticity gain during the first habit repetitions and a decreasing growth when automaticity approaches the habit’s inherent automaticity maximum. An ICC analysis showed that 78% of the total automaticity variance can be accounted for by person level (Level 2) variance, which indicates inter-individual differences in automatization. This shows the importance of modeling the given data hierarchically.

#### Automatization by Habit Repetition

To test H1a, a simple model with random slopes, random intercepts and habit repetition as fixed effect was fitted to the data (see Table 1, Model 1). As expected, habit repetition significantly predicted automaticity, *b* = 0.074 (*SE* = 0.008), *t*(58.75) = 9.09, *p* < 0.001. Intercept and slope varied significantly over participants, indicating that the initial value as well as the growth slope of automaticity differs over persons.

#### Decreasing Automaticity Growth

To test H1b, habit repetition was squared and added as a new predictor to Model 1, resulting in Model 2 (see Table 1, Model 2). As expected, habit repetition squared significantly negatively predicted automaticity, *b* = −0.001 (*SE* = < 0.001), *t*(2489.47) = −9.74, *p* < 0.001. The seemingly small, negative coefficient only marginally influences automaticity in the low habit repetition area, but, due to its quadratic nature, lowers the predicted automaticity considerably in the higher repetition area. This term replicates the asymptotic automaticity growth found by Lally et al. (2010).

#### Habit Pausing

When time is modeled via habit repetition, the model is blind to possible pauses between the logged repetitions. For example, between a participant’s habit repetition 3 and 4 could have been 2 days of not doing her habit, while between habit repetition 8 and 9 there might have been no pause. So it was tested if pausing the habit for one or more days affected the process of habit automatization to find out if there is a need to control for this condition. In the 2,483 habit repetitions after the first one, 530 (21.3%) were preceded by a pause of a day or more where no data had been logged. Pauses of more than 1 day were very rare (see preliminary results above). Habit pause was added to Model 2 as the days of pause before a habit repetition (0 = no pause, 1 = one day pause, 2 = two days pause etc.). Habit pause did not predict automaticity, *b* = −0.016 (*SE* = 0.013), *t*(2428.64) = −0.87, *p* = 0.383.

#### Age and Gender

To exclude influences of age and gender, these variables were added separately to Model 2. Neither age, *b* = 0.022 (*SE* = 0.039), *t*(103.50) = 0.56, *p* = 0.580, nor gender, *b* = −0.014 (*SE* = 0.459), *t*(93.72) = −0.030, *p* = 0.976, influenced habit automatization.

### Want Conflicts and Motivational Interference During Habit Formation

Hypotheses H2a-b aimed at showing that the strength of a habit, that is its automaticity, predicts reduced WCs and MI. The ICCs for the dependent variables indicated that 54% (WCs), respectively 44% (MI) of the total variance can be accounted for by person level variance. To control for the alternative explanation of low (high) WC/MI causing the habit to feel more (less) automatic in the moment, we included automaticity as a time lagged predictor. Leveraging the longitudinal design of the data, the automaticity score from the last habit repetition (t-1) is used to predict WC/MI in the current habit repetition (t).

#### Automaticity and Want Conflicts

To test H2a, a baseline model (Model 5) with habit repetition as the only predictor to model time and WC as the dependent variable was fitted to the data (see Table 2, Model 5). Habit repetition significantly negatively predicted WCs, *b* = −0.049 (*SE* = 0.009), *t*(57.03) = −5.73, *p* < 0.001. In a second step, automaticity_*t–*__1_ was added to Model 5 as an additional predictor resulting in Model 6 (see Table 2, Model 6). As expected, automaticity_*t–*__1_ significantly negatively predicted WCs, *b* = −0.200 (*SE* = 0.041), *t*(1765.59) = −4.87, *p* ≤ 0.001.

#### Automaticity and Motivational Interference

To test H2b, a baseline model (Model 7) with habit repetition as the only predictor to model time and MI as the dependent variable was fitted to the data (see Table 2, Model 7). Habit repetition significantly negatively predicted MI, *b* = −0.049 (*SE* = 0.007), *t*(60.19) = −7.52, *p* < 0.001. In a second step, automaticity_*t–*__1_ was added to Model 7 as an additional predictor resulting in Model 8 (see Table 2, Model 8). As expected, automaticity significantly negatively predicted MI, *b* = −0.100 (*SE* = 0.030), *t*(1439.97) = −3.37, *p* < 0.001.

#### Age and Gender

To exclude influences of age and gender, these variables were added separately to Model 5 (WC) and Model 7 (MI). Neither age, *b* = 0.034 (*SE* = 0.052), *t*(121.17) = 0.66, *p* = 0.509, nor gender, *b* = −0.743 (*SE* = 0.572), *t*(100.73) = −1.30, *p* = 0.197, influenced the experience of WCs. Furthermore, neither age, *b* = 0.035 (*SE* = 0.033), *t*(120.91) = 1.07, *p* = 0.288, nor gender, *b* = −0.419 (*SE* = 0.362), *t*(103.23) = −1.16, *p* = 0.250, influenced the experience of MI.

### Self-Control

H3a-c aimed at testing general study habit strength as a partial mediator for the effect of self-control on automaticity. H3d-e aimed at testing the influence of self-control on the experience of WCs and MI during habit performance.

#### Mediation on Aggregated Automaticity

**FIGURE 2:**
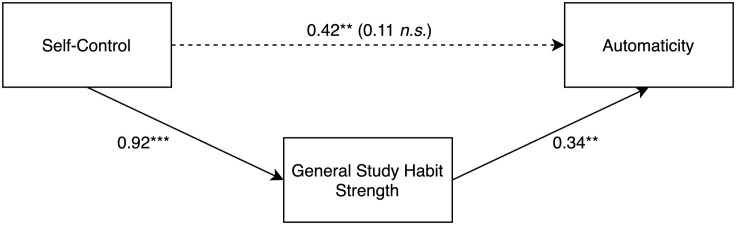
Person level mediation analysis with unstandardized regression coefficients of the effect of self-control on automaticity through general study habit strength. The first coefficient on the path from self-control to automaticity represents the total effect without the mediator; the coefficient in parentheses on this path represents the direct effect with the mediator included in the model. **p < 0.01. ***p < 0.001.

The total effect of self-control on aggregated automaticity (H3a) was *b* = 0.42 (*SE* = 0.14), *p* < 0.01. The effect of self-control on general study habit strength was *b* = 0.92 (*SE* = 0.12), *p* < 0.001, and the effect of general study habit strength on automaticity (H3b) was *b* = 0.34 (*SE* = 0.12), *p* < 0.01. The direct effect of self-control on automaticity was no longer significant after controlling for the mediator, *b* = 0.11 (*SE* = 0.18, *CI* = −0.24,0.47), *p* = 0.52, indicating an unexpected full mediation (see Figure 2). Approximately 16% of the variance in automaticity was accounted for by the predictors. The indirect effect of self-control on automaticity was estimated by bootstrapping with 5000 samples and was significant, *b* = 0.31 (*SE* = 0.11, *CI* = 0.11,0.52). H3a-b are supported by these results, while H3c has to be rejected because only a partial mediation was expected, i.e., it was assumed that self-control would still have unique predictive power after controlling for the mediator, and not a full mediation as indicated by the data.

#### Self-Control on Automaticity in MLM

To make sure that self-control did not simply increase mean automaticity by increasing habit repetition, it was tested again using multilevel modeling. The Level 2 (person level) trait variable self-control was added to Model 2, resulting in Model 3 (see Table 1, Model 3). As expected, self-control significantly positively predicted automaticity even when habit repetition was controlled, *b* = 0.428 (*SE* = 0.128), *t*(90.97) = 3.35, *p* < 0.001.

Adding general study habit strength as a Level 2 variable to Model 3 resulted in the previously found mediation pattern (see Table 1, Model 4): Self-control lost its predictive power, *b* = 0.108 (*SE* = 0.158), *t*(93.39) = 0.69, *p* = 0.493, while general study habit strength significantly predicted automaticity, *b* = 0.345 (*SE* = 0.108), *t*(92.74) = 3.20, *p* < 0.01.

#### Self-Control and Want Conflicts

To test H3d, self-control was added as a predictor to Model 5. Unexpectedly, self-control did not predict WCs, *b* = 0.056 (*SE* = 0.160), *t*(97.52) = 0.35, *p* = 0.726.

#### Self-Control and Motivational Interference

To test H3e, self-control was added as a predictor to Model 7. Unexpectedly, self-control did not predict MI, *b* = 0.079 (*SE* = 0.104), *t*(89.94) = 0.75, *p* = 0.500.

## Discussion

To our knowledge, this study is the first to document the process of building new study habits and prove reduced motivational impairments – WCs and MI – with longitudinal data. A typical habit forming pattern could be replicated from Lally et al. (2010): Habit strength (i.e., automaticity) increases with habit repetitions but this growth wanes asymptotically approaching its individual automaticity maximum with increasing habit repetitions. The positive influence of self-control on automaticity was fully mediated by general study habit strength. With increasing automaticity by habit repetitions, WCs as well as MI were significantly reduced. The genesis of new study habits and its beneficial motivational effects could be showcased in this study.

### Intentional Study Habit Building Works

Study habit building worked: The study habits became more automatic with repetitions and approached their individual maxima with asymptotically decreasing automaticity gain. In this study, habits were built *intentionally*. Intention and habit are two distinct factors that influence behavior (Ouellette and Wood, 1998; Verplanken and Aarts, 1999). In the beginning of the habit building process, intention is needed to initialize the habit building process and is the dominant factor in driving study behavior. The students had to make a conscious decision for studying every day. With habit repetitions (i.e., increasing automaticity), behavioral control shifts in favor for habitual control, lessening the influence of intention (Lally and Gardner, 2013). The students gradually start the learning process when encountering their instigation cue because that is what they normally do in this situation without having to make a conscious decision for or against it every time.

However, as stated in the introduction, study habits are characterized by changing contents but stable contexts (e.g., learning different subject matter in the same manner), which makes them more useful as this new content is learned but also more fragile as the content part of the habit can never be fully automized as the brain has to adapt to the new content in every repetition. Nevertheless, the participants’ new study habits became more automatic with each repetition, suggesting that content independent parts of the habit could be automized giving the learning activity a strong behavioral frame. This might be the habit’s starting routine, more precisely the connection between the habit instigation cue (e.g., having finished lunch) and the first sub-behavior of the habit execution (e.g., preparing the learning material), or individual sub-behaviors of each habit that can be adapted to new contents (e.g., writing a key fact on a flash card). As each individually defined habit may have a varying degree of those content independent, automizable parts, the significant random slopes in all four automaticity models (Models 1–4) come as no surprise and are likely to partially represent this variability. The strongly varying intercept of all three automaticity models can be explained in the same manner. If a new study habit contains a lot of content independent (or easily adjustable) sub-behaviors, it is very likely that these parts have already been used in previous habitual behavior and thus might bring a certain baseline automaticity to the newly defined study habit. For example, if a student has developed an individual way of summarizing key takeaways, she might bring this and other already somewhat automatic, pre-trained sub-behaviors to the newly developing, higher order study habit, resulting in a more automized behavioral sequence that will hopefully over time form a consistent habit. However, this conclusion is merely speculative and measures for the ratio of fixed and flexible parts of habits as well as for pre-trained sub-behaviors are needed to address this aspect empirically. In habit research, the chaining of multiple sub-behaviors is often addressed more broadly as behavioral complexity or difficulty (e.g., Wood et al., 2002; Verplanken, 2006) that makes it more difficult to reach high levels of automaticity in emerging habits. Verplanken (2006) may be right when he makes the point that habits cannot be reduced to the frequency of occurrence, but need to be understood as more complex mental constructs with features such as automaticity that may vary due to more factors than only behavioral repetition.

In this study, the habit building process was supported by a specifically developed mobile application that helped defining the new study habit, tracking data, reminding and giving feedback about the automaticity growth over time. That way, using the app probably became a part of the habit itself. The reminder might have acted as the instigation cue for starting and the app had to be used to enter data after each repetition. The mere thought of having to report one’s experience during the habit repetition might have increased the likelihood for compliance. Even checking the current automaticity might have become a part of the habit execution for many participants. This feedback on one’s performance might actually have acted as a contingent reward for completing a habit repetition (Henderlong and Lepper, 2002). So this app-based habit building approach is not merely about convenient event sampling, but about a new comprehensive approach to support intentional habit building.

### Habits Reduce Want Conflicts and Motivational Interference

Motivational conflicts and its consequence in the form of MI is a ubiquitous phenomenon students experience during studying (Grund et al., 2015b). Automizing a learning sequence – building a study habit – seems to be a viable intervention one can consciously implement to reduce MI during studying. Considering the Rubicon model of self-regulated behavior, there are two critical spots the automatization of study behavior is likely to have an impact on the probability of MI and motivational conflicts. Firstly, the stronger the connection between the habit instigation cue and the first sub-behavior of a habit, the easier it is to start the desired sequence (crossing the Rubicon). The quicker the decision in the pre-actional phase is made for a certain activity (e.g., learning Spanish before going to bed), the less pondering about alternative activities (e.g., watching Netflix) is to be expected. With less activation of attractive alternative activities, the experience of motivational conflicts is less likely during the actional phase (Fries and Dietz, 2007). Secondly, behavioral automatization connects the sub-behaviors of a habit, which in sum represent the activity of the actional phase, more tightly, making this behavioral sequence more consistent and leaving less space for MI through non-related activities. During the first habit repetitions of a not yet automized study habit, a student has to make many decisions on how to solve different kinds of problems, which is likely to result in decision fatigue if the several viable options have to be considered every time (Vohs et al., 2008; Pocheptsova et al., 2009). Best practices are likely to emerge over time that are chosen as a default without a lot of pondering if a certain problem comes up again in future habit repetitions.

Habit building is a valuable approach to reducing WC/MI as it is an intentional process that can be harnessed by everyone independently of fixed personality variables. The tendency to automize often repeated behavior by the brain is an overarching principle of human adaption: It is highly adaptive to reduce the energy, in this case mental effort, that is required to perform these stable behavioral patterns (Hodgson, 2010). This happens unconsciously. But by proper planning and precise habit definition, it is possible to evoke this useful tendency. On the other hand, there is one trait that plays a major role in both, habit building and the experience of MI: Self-control.

### Self-Control Facilitates Self-Regulation Indirectly via Behavioral Automaticity

Self-control is a central variable as well as in the realm of motivational conflicts and MI in learning processes (Grund and Fries, 2014; Grund et al., 2015a) as in the realm of habits (de Ridder et al., 2012; Neal et al., 2013; Galla and Duckworth, 2015). In our first set of self-control hypotheses (H3a–c), we tested a mediation of the effect of self-control on automaticity through general study habit strength. We assumed that self-control increases automaticity in the habit building process in two ways: Directly and indirectly. The direct path, a main effect of self-control on automaticity, could not be found after controlling for general study habit strength. Concerning the indirect path however, general study habit strength fully mediated the effect of self-control. We think of this indirect path as the transmission of already automized fractions from past study habits that are used as the building blocks of the new habit. If highly self-controlled people start the habit building process with more “pre-automized” building blocks for their learning habit and thus are more likely to build stable habits, these products of past self-control might actually be a more direct supporting factor in habit building than the ability to resist impulses in itself. If less impulses come up in the learning process to begin with, less self-control is needed overall.

Unexpectedly, self-control had no relation to the experience of motivational impairments during the tracked learning activities. In a cross sectional study with an imagined motivational conflict while studying, this trait predicted less MI (e.g., Galla and Duckworth, 2015, study 2) as well as less WCs during studying in experience sampling (Grund et al., 2015a). We could not replicate a direct effect of self-control on WC/MI, which would represent a volitional inhibition of impulses during studying (i.e., the actional phase of the Rubicon model). On the other hand, self-control has indeed this unintuitive relationship to its own exertion: The stronger it is, the less it is used (Grund and Carstens, 2019), which is partially mediated by habit strength (Galla and Duckworth, 2015, study 1). Hofmann et al. (2012) found in an experience sampling study that self-control did not influence how people could resist in situations of temptation but it predicted how often people would get into situations of temptation in the first place. In line with our findings, Hofmann et al. suggest that self-control operates via adaptive habits.

We found that self-control fosters habit building and thus automizing behavior. Stronger automaticity of study behavior in turn reduced WC/MI. A positive effect of self-control on different desirable outcomes such as good grades, college persistence, classroom engagement, homework completion, regular meditation, exercising, getting consistent sleep, eating healthy snacks, less MI during studying, less effortful inhibition (Galla and Duckworth, 2015) and more desired behavior especially in the realm of work and school has repeatedly been shown to be mediated by habits (de Ridder et al., 2012). Furthermore, it has to be noted that we related a trait level measure of self-control with *in situ* experiences of WC/MI in a specific activity, while other cross sectional studies, where an association between self-control and WC/MI was found, used imagined motivational conflicts (Galla and Duckworth, 2015, study 2) or self-report measures of motivational conflicts where the participants need to sample their own memory and make guesses about an aggregated average motivational conflict value over a broader array of activities from the past (Kuhnle et al., 2010). These cross sectional measurement situations are prone to measurement artifacts due to selective memory effects or mood during the measurement time. Likewise, in an experience sampling study showing a connection between self-control and WCs (Grund et al., 2015a), experiences were sampled at random times scattered over the day. Following the habit-as-a-mediator-logic, self-control leading to less experienced WCs might have been caused by a higher probability of sampling experiences during automized behavior, which is more prevalent in highly self-controlled individuals.

So according to our findings, the role of self-control for learning is mostly important for building study habits. Strong habits are more likely to be repeated regularly with less motivational impairments in the form of WC/MI and are thus a very effective way to achieve long-term goals.

### Study Limitations

One core assumption concerning the formation of new study habits we make here is that a learner draws from her past experience when building new habits. Well practiced (“pre-automized”), parts of another study habit are likely to be implemented as building blocks for the new study habit. Following this logic, a “new” habit with a relatively large proportion of already automized building blocks from old habits should have a higher automaticity to begin with, which should in part account for the random intercept variance in the automaticity models. A measure to determine the exact proportion of old habit in a new habit would be necessary to test this assumption thoroughly. The measurement of general study habit strength, as used in this study, constitutes an economic proxy for this. However, with this measure it cannot be determined how many of the old building blocks have actually been used for the construction of a new habit, but only how large the pool of building blocks is and how automized they are in general.

The ecological validity of an *in situ* event sampling approach is generally one of its distinct advantages. However, there are still two issues with the study context in which this habit forming data has been collected. Firstly, it was salient to the participants that their habit performance is part of a study, in which consistent habit repetition is expected by the researcher. Secondly, the course credit that a majority of the participants received for participation was dependent on compliance, adding an extra extrinsic reward that would normally not be part of the habit building process.

Finally, our findings are derived from a very specific population: Mainly German, young, female psychology students took part in the study and all of them were iPhone users due to technical constraint. This might restrict the generalizability of the findings.

## Conclusion

Our research provided evidence for a viable way to reduce WC/MI while studying: Intentional, app aided study habit building. By the use of event sampling, we could observe the genesis of a new habit and its psychometric changes over time to showcase a strong link between automaticity and reduced WC/MI. The role of trait self-control in this process was surprising in so far as it predicted habit formation but not WC/MI in the moment of study habit performance. The way self-control impacts the experience of WC/MI seems so be rather indirectly by enabling more efficient automizing of behavior than directly by effortful inhibition of impulses during a given task. The fact that good habits – useful and stable patterns of automized behavior that are performed in stable contexts – are, firstly, more resilient to motivational impairments and thus more likely to be productive toward a desired longterm outcome and, secondly, can in all probability be used to build similar future habits more efficiently, makes them very valuable mental assets.

## Data Availability Statement

The datasets generated for this study are available on request to the corresponding author.

## Ethics Statement

The studies involving human participants were reviewed and approved by the Bielefeld University, Ethics Committee. Written informed consent for participation was not required for this study in accordance with the national legislation and the institutional requirements. Written informed consent was inferred through the completion of the pretest questionnaire.

## Author Contributions

MS developed the idea and study design, created the iPhone application, conducted the data acquisition, performed the statistical analyses, and wrote the manuscript. AG and SF contributed to the final version of the manuscript and provided critical feedback, which helped shaping the theoretical foundation of the paper. SF supervised the project.

## Conflict of Interest

The authors declare that the research was conducted in the absence of any commercial or financial relationships that could be construed as a potential conflict of interest.

## Footnotes

1 In 19.0% of the measurement occasions, students reported feeling they should do something else (i.e., should conflict). However, these should conflicts were accompanied by WCs 83.6% of the time, leaving only 3.1% of “pure” should conflicts while studying.
